# Further study of Late Devonian seed plant *Cosmosperma polyloba*: its reconstruction and evolutionary significance

**DOI:** 10.1186/s12862-017-0992-1

**Published:** 2017-06-26

**Authors:** Le Liu, Deming Wang, Meicen Meng, Jinzhuang Xue

**Affiliations:** 1 College of Geoscience and Surveying Engineering, China University of Mining and Technology (Beijing), Beijing, 100083 China; 2 Key Laboratory of Orogenic Belts and Crustal Evolution, Department of Geology, Peking University, Beijing, 100871 China; 3 Science Press, China Science Publishing & Media Ltd., Beijing, 100717 China

**Keywords:** *Cosmosperma polyloba*, Frond, Ovule, Pollen organ, Seed plant, Late Devonian, Wutong Formation, South China

## Abstract

**Background:**

The earliest seed plants in the Late Devonian (Famennian) are abundant and well known. However, most of them lack information regarding the frond system and reconstruction. *Cosmosperma polyloba* represents the first Devonian ovule in China and East Asia, and its cupules, isolated synangiate pollen organs and pinnules have been studied in the preceding years.

**Results:**

New fossils of *Cosmosperma* were obtained from the type locality, i.e. the Leigutai Member of the Wutong Formation in Fanwan Village, Changxing County, Zhejiang Province, South China. The collection illustrates stems and fronds extensively covered in prickles, as well as fertile portions including uniovulate cupules and anisotomous branches bearing synangiate pollen organs. The stems are unbranched and bear fronds helically. Fronds are dimorphic, displaying bifurcate and trifurcate types, with the latter possibly connected to fertile rachises terminated by pollen organs. Tertiary and quaternary rachises possessing pinnules are arranged alternately (pinnately). The cupule is uniovulate and the ovule has four linear integumentary lobes fused in basal 1/3. The striations on the stems and rachises may indicate a *Sparganum*-type cortex.

**Conclusions:**

*Cosmosperma* further demonstrates diversification of frond branching patterns in the earliest seed plants. The less-fused cupule and integument of this plant are considered primitive among Devonian spermatophytes with uniovulate cupules. We tentatively reconstructed *Cosmosperma* with an upright, semi-self-supporting habit, and the prickles along stems and frond rachises were interpreted as characteristics facilitating supporting rather than defensive structures.

**Electronic supplementary material:**

The online version of this article (doi:10.1186/s12862-017-0992-1) contains supplementary material, which is available to authorized users.

## Backgrounds

Many ovules have been reported from the Upper Devonian (Famennian) of Europe, North America and China, and they indicate the first major radiation of seed plants or spermatophytes [1–4]. Pollen organs also add to our knowledge about these earliest spermatophytes, although they are usually detached from the ovules or fronds [3, 5–10]. Despite the abundance of fertile structures (>20 genera of ovules and pollen organs) in the Late Devonian, the frond morphology and overall architecture is only known for a few seed plant taxa.

South China was an isolated crustal plate with great plant diversity in the Devonian [11–13]. However, seed plant were only recently found in the Late Devonian of this plate, displaying cupulate ovules, pollen organs and stem anatomy [3, 4, 8–10, 13]. These findings suggest that China is an important area for understanding the early evolution of seed plants. Among them, *Cosmosperma polyloba* represents the first Devonian ovules known from China and East Asia that are associated with pollen organs and pinnules [3], while the details of the ovules are unclear due to poor preservation. Based on new specimens from the type locality, we now emend the diagnoses of *Cosmosperma*, compare its frond morphology to related taxa and provide further information regarding its overall architecture. The entire plant is reconstructed and its evolutionary significance is discussed.

## Material and Methods

Over 100 new specimens of *Cosmosperma polyloba* were obtained from the Wutong (Wutung) Formation in a quarry near Fanwan Village, Hongqiao Town, Changxing County, Zhejiang Province, China. The information regarding the locality and stratigraphy has been provided in recent studies [3, 14, 15]. At the Fanwan section, the Wutong Formation is divided into the Guanshan Member, with quartz sandstone and conglomerate, and the overlying Leigutai Member, with interbedded quartz sandstone and mudstone. The fossil plant occurs at the 13th bed of the Wutong Formation (in the Leigutai Member), i.e. the same bed from which former specimens of *Cosmosperma* and strobili of lycopsid *Changxingia* sp. were collected [3, 15]. The LC (*Knoxisporites literatus*-*Reticulatisporites cancellatus*) spore assemblage suggests that the upper part of the Leigutai Member is of the latest Famennian age [16].

In siltstone with tiny crystals of quartz and white micas, the plant is preserved as dark-brown compressions and impressions, displaying great contrast to the yellowish matrix. Steel needles were applied to expose the plant morphology and a digital camera and a stereoscope were used for photographs. All the specimens are housed at the Department of Geology, Peking University, Beijing, China.

### Systematics

**Division** Spermatophyta sensu Rothwell and Serbet 1994

**Class** Lagenospermopsida sensu Cleal 1994

**Order and Family** Incertae sedis

**Genus** *Cosmosperma* Wang et al. 2014 emend.

**Emended diagnosis:** (emended and additional generic characters are in brackets).

[Seed plant with unbranched stems bearing dimorphic fronds, dichotomized fertile rachises terminated by synangiate pollen organs, and cupulate ovules. Fronds with a swollen pulvinus-shaped base. Majority of fronds bifurcate, with primary rachis dichotomizing into two secondary rachises. The other fronds trifurcate, with primary rachis ended by two subopposite secondary rachises and one median rachis. Tertiary rachises and ultimate pinnae (with quaternary rachis) borne alternately and pinnately.] Nonlaminate pinnules planate, highly dissected and alternately arranged on [quaternary rachis]. Pollen organs synangiate, with each terminating a stalk and consisting of [four] to eight elongate microsporangia that are basally fused and distally free. Uniovulate cupules with [up to approximately 16 tips]; cupule [tips] free for a length of half to two thirds that of cupules. [Ovule connected to cupule by a short stalk. Four linear integumentary lobes fused in the basal 1/3. Tiny conical prickles occurring on stems, four orders of frond rachises, cupules and fertile rachises.]

**Type species** *Cosmosperma polyloba* Wang et al. 2014 emend.

**Holotype:** PKUB13401a, b ([3], original Fig. 2b).

**Fig. 1:**
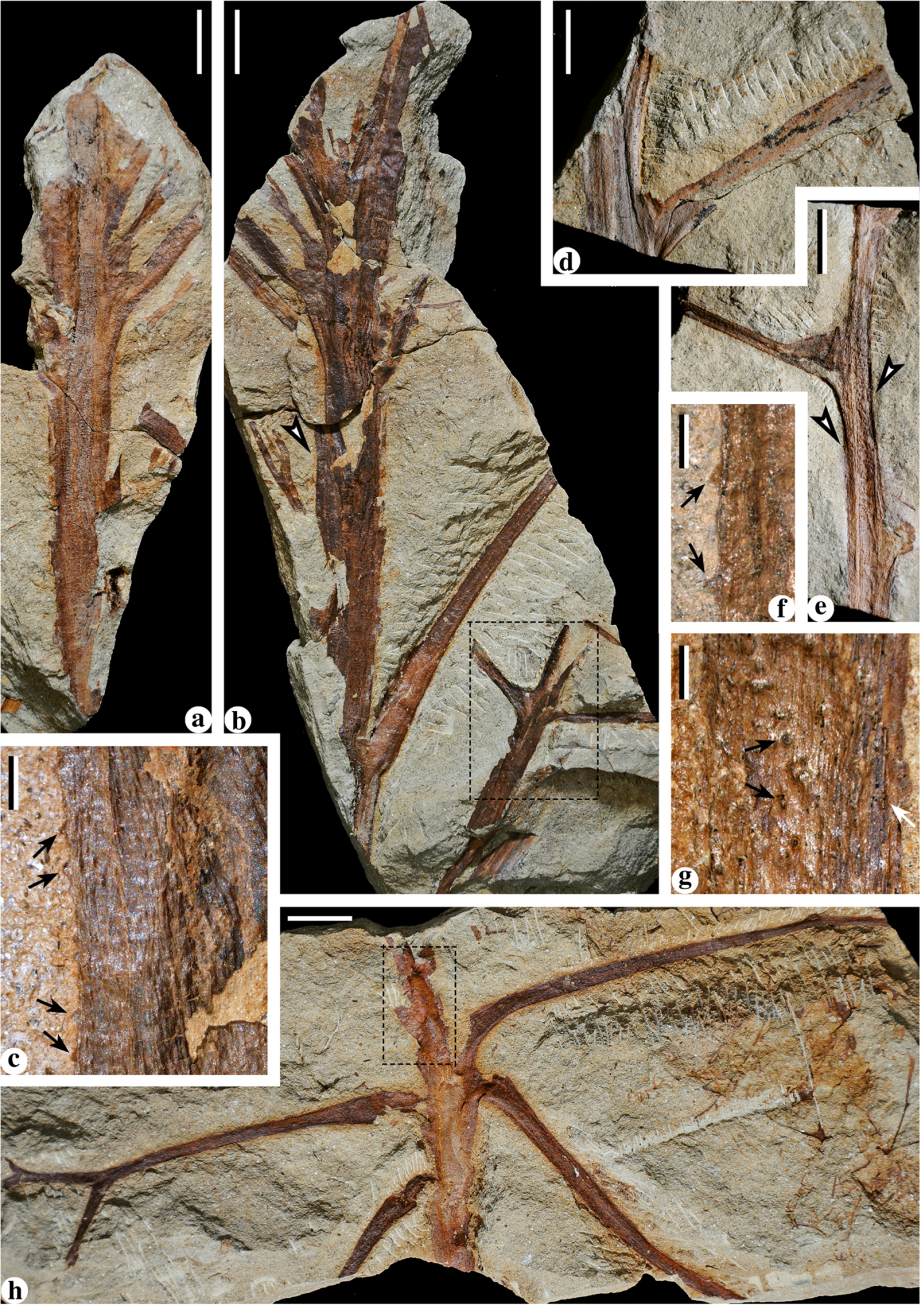
Stems of *Cosmosperma polyloba*. **a** Stem with primary rachises. PKUB13501a. Scale bar = 20 mm. **b** Combined figure of two counterparts of specimen shown in Fig. 1 **a**, exhibiting stem with primary rachises. *Arrow* indicating portion enlarged in Fig. 1 **c**. PKUB13501b (part in *dashed box*), PKUB13501c. Scale bar = 20 mm. **c** Enlargement of Fig. 1 (**b**, *arrow*), showing conical prickles (*arrows*) and parallel vertical striations on the stem. Scale bar = 2 mm. **d** Stem with the widest primary rachis. PKUB13502. Scale bar = 20 mm. **e** Stem and helically arranged primary rachises with swollen bases. *Arrows* indicating portions enlarged in Fig. 1 **f**, **g**. PKUB13503. Scale bar = 20 mm. **f** Enlargement of Fig. 1 (**e**, *left arrow*), showing conical prickles (*arrows*). Scale bar = 2 mm. **g** Enlargement of Fig. 1 (**e**, *right arrow*), showing parallel vertical striations (*white arrow*) and conical prickles preserved as pit-like impressions (*black arrows*). Scale bar = 2 mm. **h** Combined figure of part and counterpart of one specimen, showing a stem with primary rachises arranged in irregular helix. PKUB13504a, PKUB13504b (*dashed box*). Scale bar = 20 mm

**Fig. 2:**
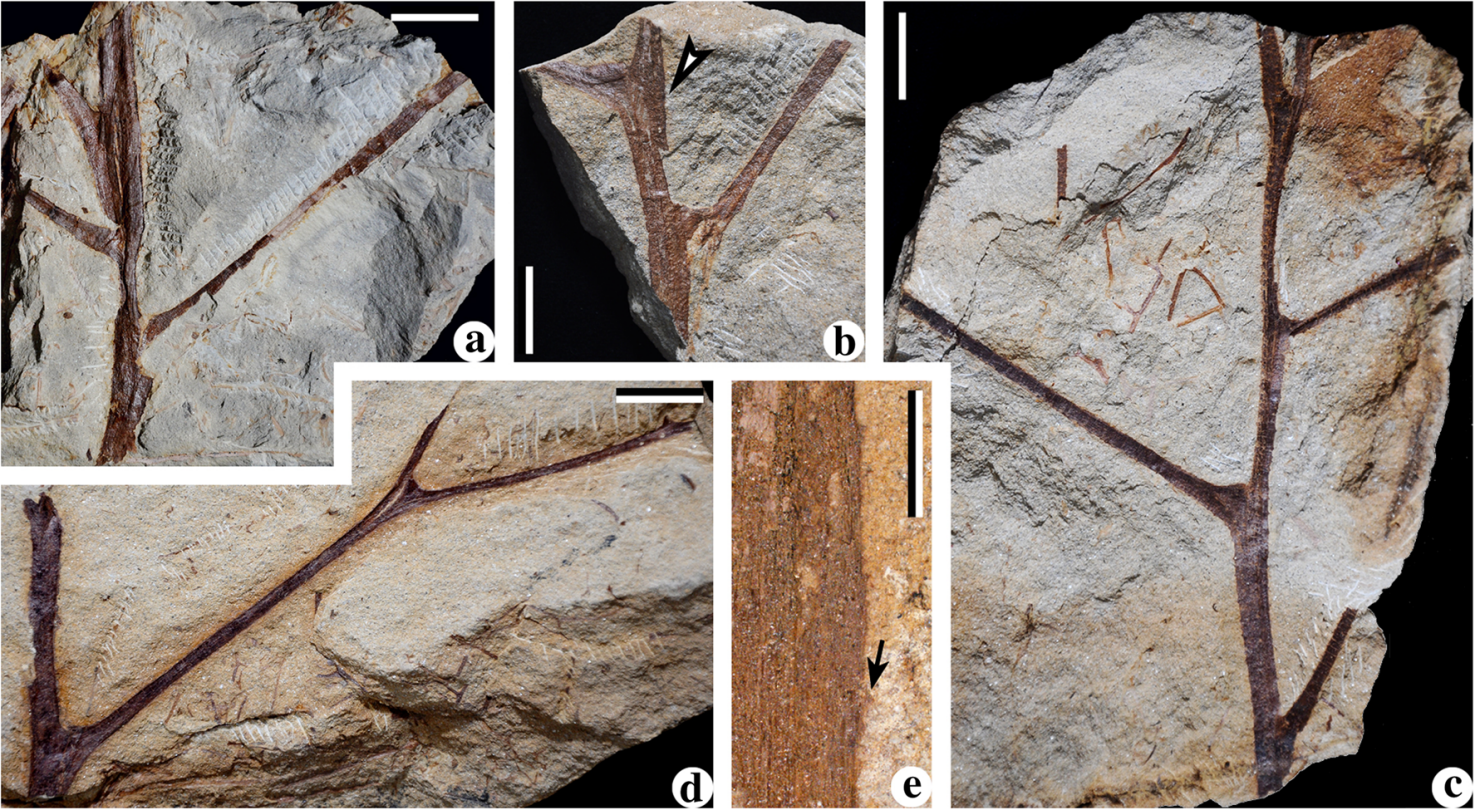
Stems of *Cosmosperma polyloba*. **a**-**c** Stems illustrating helically arranged primary rachises with swollen bases. *Arrow* in Fig. 2 **b** indicating portion enlarged in Fig. 2 **e**. PKUB13505-PKUB13507. Scale bars = 20 mm. **d** Stem with one dichotomous frond rachis. PKUB13508. Scale bar = 20 mm. **e** Enlargement of Fig. 2 (**b**, *arrow*), showing conical prickles (*arrow*) and parallel vertical striations. Scale bar = 5 mm

**Specimens examined herein:** PKUB13501-PKUB13517 (Figs. 1, 2, 4, 5, 6 and 7).

**Repository:** Department of Geology, Peking University, Beijing, China.

**Locality & horizon:** Fanwan Village, Hongqiao Town, Changxing County, Zhejiang Province, China; Leigutai Member of Wutong Formation, Upper Devonian (Famennian).

**Emended diagnosis:** (Emended and additional specific characters are in brackets).

As for generic diagnosis. [Stems up to 25.9 cm long and 2.2 cm wide, with internodes 0.6–6.2 cm long. Fronds departing at 40–70°. Primary rachises 10.3–21.2 cm long and 3.0–12 mm wide. The secondary rachises are up to 14.3 cm long and 1.8–3.6 mm wide. Median rachises of trifurcate fronds up to 10.9 cm long and ca. 4.0 mm wide. Tertiary rachises up to 10.9 cm long and 1.7–2.9 mm wide. First tertiary rachises occurring on outside of frond. Ultimate pinna] up to 54 mm long and 28 mm wide, with [quaternary rachis] about 0.7 mm wide; pinnules [6.0]–13.3 mm long and [3.0]–13.0 mm wide, borne at angles of 70–90°, and consisting of one terminal unit and four alternately arranged lateral units. Pinnule units 4.2–7.2 mm long and 2.8–8.3 mm wide, equally dichotomous for one to three times. [Fertile rachises dichotomizing 3–6 times at 50–120°, with intervals between adjacent bifurcating points 1.4–19.3 mm long and 0.3–1.2 mm wide.] Pollen organs borne in pairs, 2.2–[2.5] mm long and [2.0]–2.9 mm wide, with stalks 1.0 mm long and 0.2–0.3 mm wide; microsporangia 2.3 mm long and [0.3]–0.7 mm wide, and distally tapered. Cupules [5.3]–8.8 mm long and [3.0]–9.0 mm wide; pedicels 1.0 mm long and 0.4 mm wide; ovules 3.7–[4.7] mm long and 1.6–[2.2] mm wide; [ovule stalk ca. 0.2 mm long and ca. 0.5 mm wide; integumentary lobes ca. 3.8 mm long and ca. 0.5 mm wide. Prickles on stems and proximal parts of fronds, ca. 0.3 mm long and ca. 0.5 mm wide at the base; those on cupules and distal fertile rachises, ca. 0.2 mm long and ca. 0.3 mm wide at the base].

## Results

### General morphology

**Fig. 3:**
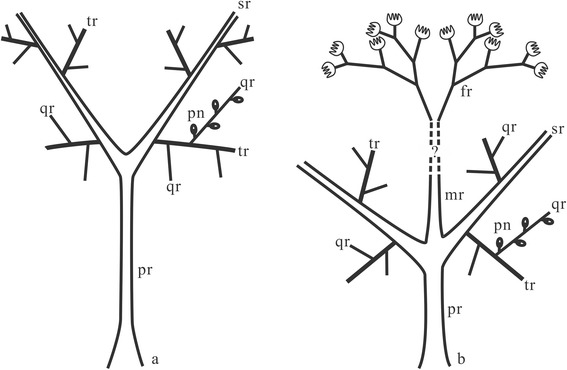
Interpretative diagram showing architecture of bifurcate (**a**) and trifurcate (**b**) fronds of *Cosmosperma polyloba*. The connection between trifurcate frond and fertile rachises bearing synangia (*dashed lines with a question mark*) is speculative. Abbreviations: pr, primary rachis; sr, secondary rachis; mr, median rachis; tr, tertiary rachis; qr., quaternary rachis; fr, fertile rachis; pn, pinnule

**Fig. 4:**
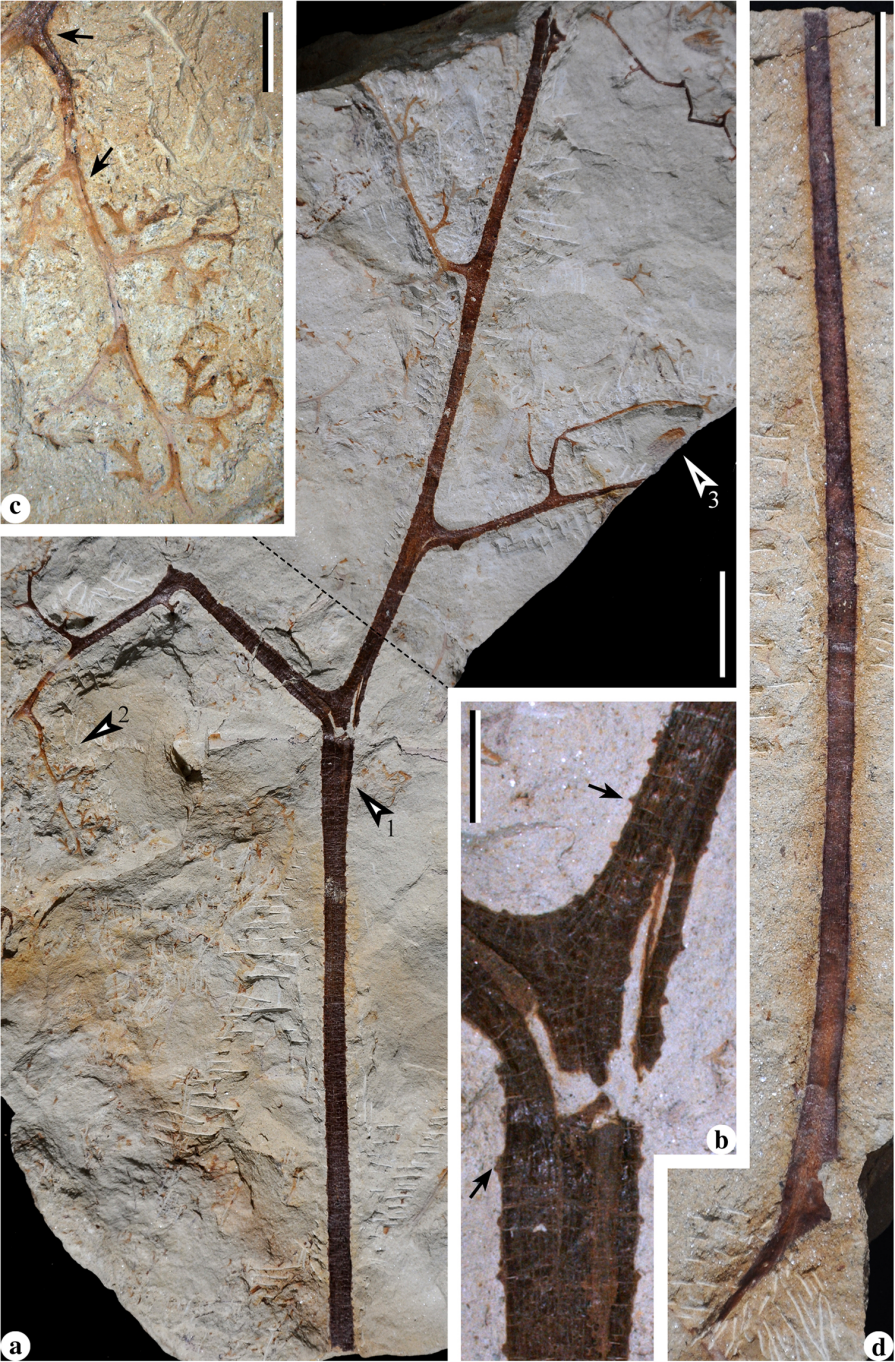
Bifurcate fronds of *Cosmosperma polyloba*. **a** Combined figure of part and counterpart of one specimen, exhibiting frond with primary rachis ending in a dichotomy (*arrow* 1, enlarged in Fig. 4 **b**), two secondary rachises, alternately (pinnately) arranged tertiary rachises and an attached ultimate pinna (quaternary rachis and pinnules; *arrow* 2, enlarged in Fig. 4 **c**). A cupulate ovule (*arrow* 3, enlarged in Fig. 6 **d**) is associated with the frond. PKUB13509a, PKUB13509b (above the *dashed line*). Scale bar = 20 mm. **b** Enlargement of Fig. 4(**a**, *arrow* 1), showing the dichotomy of primary rachis. Note parallel vertical striations and conical prickles (*arrows*) along the primary rachis and basal part of secondary rachises. Scale bar = 5 mm. **c** Enlargement of Fig. 4 (**a**, *arrow* 2), showing one ultimate pinna with highly dissected, alternate and planate pinnules. *Arrows* indicating conical prickles along quaternary rachis. Scale bar = 5 mm. **d** Longest primary rachis with base but without distal portion preserved. PKUB13508. Scale bar = 20 mm

**Fig. 5:**
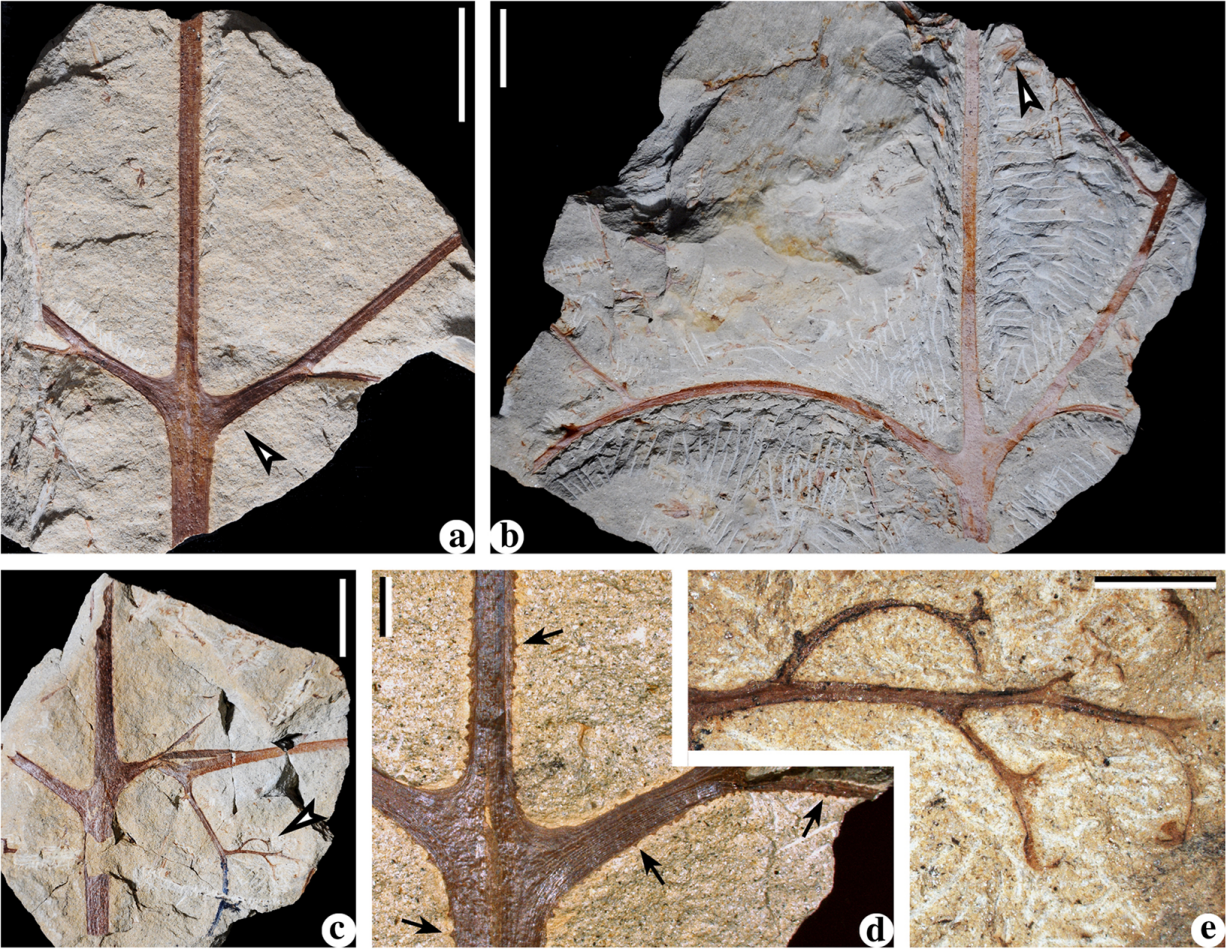
Trifurcate fronds of *Cosmosperma polyloba*. **a** Frond consisting of a primary rachis, a median and two subopposite secondary rachises and two tertiary rachises. *Arrow* indicating portion enlarged in Fig. 5(**d**). PKUB13510a. Scale bar = 20 mm. **b** Frond consisting of a primary rachis, a median and two subopposite secondary rachises and alternately borne tertiary rachises. One cupulate ovule (*arrow*, enlarged in Fig. 6 **e**) associated with the frond. PKUB13511. Scale bar = 20 mm. **c** Frond consisting of a primary rachis, a median and two subopposite secondary rachises, a tertiary rachis and an ultimate pinna. *Arrow* indicating portion enlarged in Fig. 5e. PKUB13512. Scale bar = 20 mm. **d** Enlargement of Fig. 5 (**a**, *arrow*), showing attachment of a median and two secondary rachises. Parallel vertical striations distributed along primary rachis, basal parts of median and secondary rachises. *Arrows* indicating conical prickles. Scale bar = 5 mm. **e** Enlargement of Fig. 5 (**c**, *arrow*), showing an ultimate pinna with poorly preserved pinnules. Scale bar = 5 mm

**Fig. 6:**
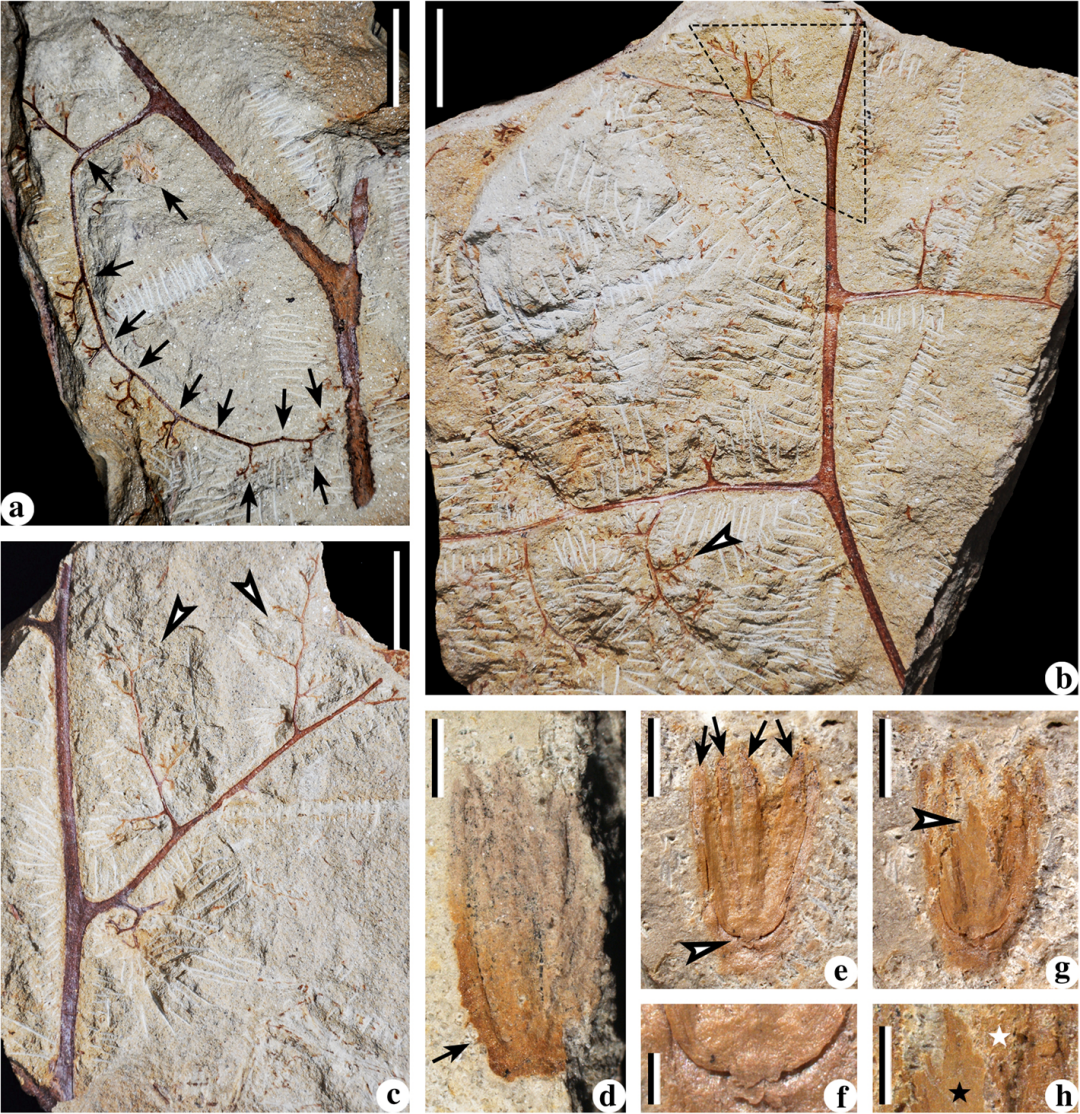
Fronds and cupulate ovules of *Cosmosperma polyloba*. **a** Frond consisting of distal part of bifurcated primary rachis, two secondary rachises, and one tertiary rachis bearing 11 ultimate pinnae (*arrows*). PKUB13513. Scale bar = 20 mm. **b** Combined figure of part and counterpart of one specimen, showing vegetative frond with secondary rachis at possible distal portion and three alternate (pinnate) tertiary rachises bearing ultimate pinnae. *Arrow* indicating portion redrawn in Additional file 2: Figure S2(d). PKUB13514a, PKUB13514b (*dashed polygon*). Scale bar = 2 cm. **c** Frond segment including secondary rachis, alternately (pinnately) borne tertiary rachises with ultimate pinnae. *Arrow* indicating portions redrawn in Additional file 2: Figure S2(b, c). PKUB13515. Scale bar = 20 mm. **d** Enlargement of cupulate ovule in Fig. 4(a, *arrow* 3), showing prickles (*arrow*) on the outer surface. Scale bar = 2 mm. **e** Enlargement of cupulate ovule in Fig. 5(b, *arrow*), showing compression of ovule with a stalk connecting the cupule (*lower arrow*, enlarged in Fig. 6 **f**) and four integumentary lobes (*upper four arrows*). Scale bar = 2 mm. **f** Enlargement of Fig. 6 (**e**, *lower arrow*), showing lower part of ovule with a short stalk. Scale bar = 0.5 mm. **g** Ovule in Fig. 6e, remnant after removal of integumentary lobes by dégagement. *Arrow* indicating portion enlarged in Fig. 6 **h**. Scale bar = 2 mm. **h** Enlargement of Fig. 6 (**g**, *arrow*), showing cupule tips (*white star*) and remnant of ovule (*black star*). Scale bar = 0.5 mm

**Fig. 7:**
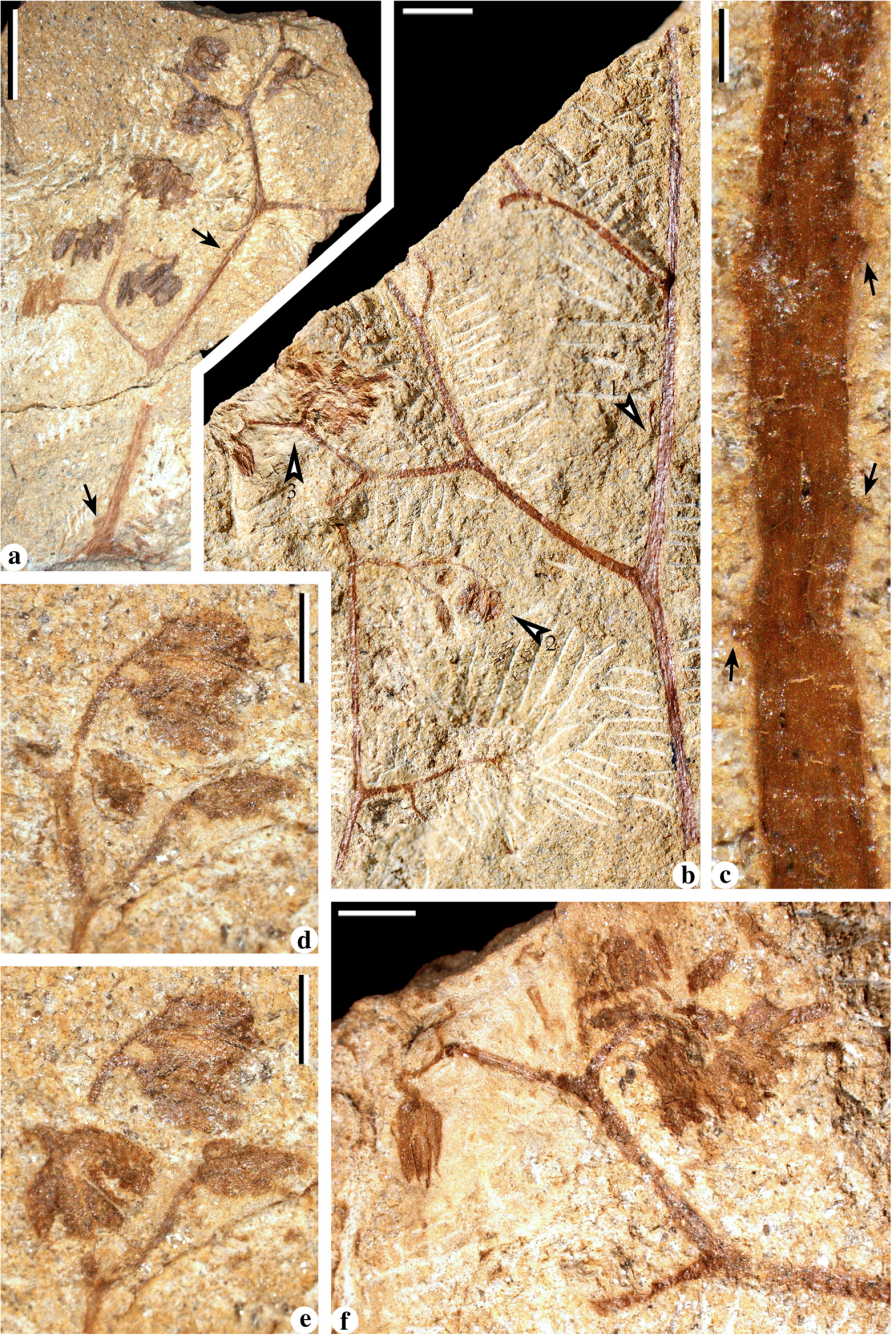
Synangiate pollen organs on fertile rachises of *Cosmosperma polyloba*. **a** Anisotomous fertile rachises with terminal pollen organs and prickles (*black arrows*). PKUB13516. Scale bar = 5 mm. **b** Two anisotomous fertile rachises with terminal pollen organs. Fertile rachises (*arrow* 1, enlarged in Fig. 7 **c** displaying sparse prickles; pollen organ (*arrow* 2, 3, enlarged in Fig. 7 **d**-**f**) born in pairs or singly. PKUB13517. Scale bar = 5 mm. **c** Enlargement of Fig. 7 (**b**, *arrow* 1), showing one fertile rachis bearing conical prickles (*black arrows*). Scale bar = 0.5 mm. **d**, **e** Enlargement of Fig. 7 (**b**, *arrow* 2). Two stages of dégagement showing pollen organs. Scale bar = 2 mm. **f** Enlargement of Fig. 7 (**b**, *arrow* 3). Pollen organs terminating bifurcated fertile rachises. Scale bar = 2 mm

Plant organs of *Cosmosperma polyloba* described here include stems (Figs. 1 and 2), dimorphic fronds (Figs. 3, 4, 5 and 6a-c), isolated cupulate ovules (Fig. 6d-h), and synangiate pollen organs terminating anisotomous fertile rachises (Fig. 7). One stem, some fronds and fertile rachises with pollen organs are represented in interpretive line-drawings (Additional files 1, 2 and 3: Figures S1–S3). Fronds are arranged in irregular helices on the stem (Figs. 1a, b, e, h and 2a–c, Additional file 1: Figure S1a) and consist of up to four orders of rachises (Figs. 4a, 5a–c and 6a–c, Additional files 1 and 2: Figures S1b–d, S2a) and pinnate pinnules (Figs. 4c and 5e, Additional file 2: Figure S2b–e). Morphological descriptors for fronds are illustrated in Fig. 3. Tiny conical prickles of different size are present on the stems (Fig. 1c, arrows, f, arrows, g, black arrows, 2e, arrow), frond rachises (Fig. 4b, arrows and Fig. 5d, arrows), cupules (Fig. 6d, arrow) and fertile rachises bearing pollen organs (Fig. 7a, arrows, c, arrows). Narrow and parallel striations occur vertically on the surface of the stems (Fig. 1c, g, white arrow; Fig. 2e), primary rachises and basal part of secondary rachises (Figs. 4b and 5d), implying the *Sparganum*-type cortex.

### Stems

Stems are 0.7–2.2 cm wide (Figs. 1 and 2) and up to 25.9 cm long (Fig. 1b). No evidence indicates that the stems are branched. The large stems (Fig. 1) suggest basal or mature parts of the plant, while the slender ones (Fig. 2) may represent the upper or immature portions. The prickles are ca. 0.3 mm long and ca. 0.5 mm wide at the base (Fig. 1c, arrows, f, arrows and Fig. 2e, arrow), and they sometimes leave pit-like impressions on the stem surface (Fig. 1, g, black arrows).

### Fronds

Along the stem, the internodal length between the attachments of two adjacent fronds ranges from 0.6–6.2 cm (Fig. 1a, b, e, h; Fig. 2a–c). The fronds depart at 40–70° (Fig. 1a, b, d, e; Fig. 2a–d), and their bases are swollen and pulvinus shaped (Figs. 1e and 2b–d). Fronds exhibit two types of division (Fig. 3), i.e. the bifurcate type (Figs. 3a and 4) and the trifurcate type (Figs. 3b and 5), which can be distinguished by the primary rachises. Most fronds are bifurcate, showing primary rachises that extend a long distance and then dichotomize at 45–70° into two secondary rachises (Figs. 1h, 2d, 4a and 6a). The trifurcate fronds possess a primary rachis that ends in two subopposite secondary rachises departing at 60–90° and a median rachis (Fig. 1b, dashed box; Fig. 5a–d). The total length of fronds is up to 24.2 cm (Fig. 4a). Primary rachises are 10.3–21.2 cm long (Figs. 2d and 4d), and are usually 3.0–4.0 mm wide, but can be up to 12 mm wide (Fig. 1d). The secondary rachises are up to 14.3 cm long and 1.8–3.6 mm wide. The median rachises of trifurcate fronds are up to 10.9 cm long and ca. 4.0 mm wide, demonstrating parallel vertical striations and tiny conical prickles (Fig. 5d). Tertiary rachises are borne alternately (Figs. 4a, 5b and 6b, c, Additional file 1: Figure S1b–d), and up to 10.9 cm long and 1.7–2.9 mm wide. Two proximal tertiary rachises are produced toward the outside of the frond, at the same distance from the base of the secondary rachis (Figs. 4a and 5a). Ultimate pinnae are mainly alternately (i.e., pinnately) arranged (Figs. 4a, 5c and 6a–c, Additional files 1 and 2: Figures S1b–d, S2a), but occasionally folded to one side (Fig. 6a) due to preservation. The quaternary rachises (ultimate pinna rachises) are up to 4.1 cm long and 0.7 mm wide (Figs. 4c, 5e and 6b, c, Additional file 2: Figure S2b–e). The amount of quaternary rachises on a single tertiary rachis is up to 11 (Fig. 6a, Additional file 2: Figure S2a). The prickles on frond rachises are ca. 0.3 mm long and ca. 0.5 mm wide at the base (Fig. 4b, c, arrows and Fig. 5d). Highly dissected but planate pinnules are alternately arranged along the quaternary rachis, and are 6.0–13.0 mm long and 3.0–10.0 mm wide (Figs. 4c, 5e and 6b, c, Additional file 2: Figure S2, b–e). Each pinnule exhibits an “axis”, with several alternately-borne lateral units and one terminal unit. These units dichotomize into several slender segments (Figs. 4c and 6b, c, Additional file 2: Figure S2b–e). The axis and the segments are ca. 0.5 mm wide.

### Cupulate ovules

Cupules are isolated, 5.3–7.7 mm long and 3.0–5.1 mm at the maximum width (Fig. 6d, e). The cupules display minute conical prickles on the outer surface (Fig. 6d, arrow) that are ca. 0.2 mm long and ca. 0.3 mm wide at the base. Each cupule possesses segments with multiple tips that are about half of the total cupule length and are ca. 0.5 mm wide. One specimen illustrates that the cupule is uniovulate (Fig. 6e). The upper part of the ovule (Fig. 6g, arrow) is dégaged to expose several cupule tips (Fig. 6h, white star), which are beneath the ovule remnant (Fig. 6h, black star). Before the dégagement, this ovule was 4.7 mm long and 2.2 mm wide, and connected to the cupule by a short stalk ca. 0.2 mm long and 0.5 mm wide (Fig. 6e, lower arrow, f). Four integumentary lobes are linear and straight (Fig. 6e, black arrows), ca. 3.8 mm long and ca. 0.5 mm wide, and fused to each other in the basal 1/3 of the ovule.

### Fertile rachises with terminal pollen organs

The fertile rachises are anisotomous and terminate in pollen organs (Fig. 7a, b, d–f; Additional file 3: Figure S3). These rachises dichotomize 3–6 times and at an angle of 50–120°, with the intervals between two adjacent bifurcating points being 1.4–19.3 mm long and 0.3–1.2 mm wide. Both length and width of the intervals reduce acropetally. Conical prickles are sparse on the branches and ca. 0.2 mm long and 0.3 mm wide at the base (Fig. 7a, c, Additional file 3: Figure S3a). Pollen organs, ca. 2.5 mm long and 2.0 mm wide, are born mainly in pairs, but sometimes singly or incompletely preserved (Fig. 7d–f; Additional file 3: Figure S3). Individual pollen organs are synangiate with basally fused microsporangia. Each synangium consists of 4–8 elongate microsporangia, which are ca. 2.3 mm long and 0.3–0.4 mm wide.

## Discussion

### Reconstruction of *Cosmosperma*

**Fig. 8:**
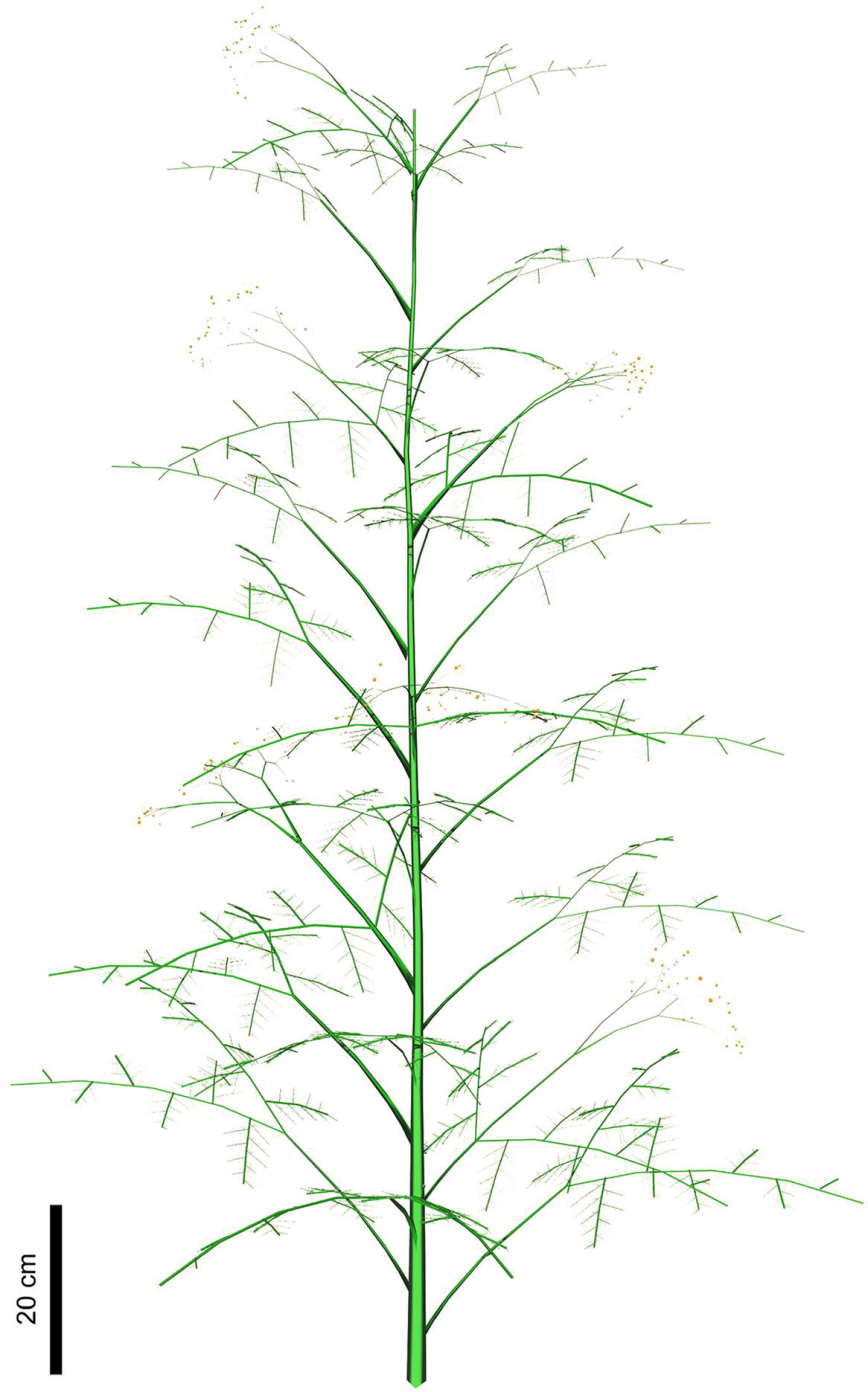
Reconstruction of *Cosmosperma polyloba*. The plant is considered to possess an upright, probably semi-self-supporting habit, with adjacent individuals entangled by their bushy, prickle-bearing fronds. Dimorphic fronds are helically arranged along stem, with bifurcate fronds in the majority, and scattered trifurcate fronds displaying median rachises; the connections between trifurcate fronds and fertile parts are speculative

Based on the specimens described above, *Cosmosperma* is characterized by unbranched stem with two types of fronds attached in irregular helices, alternately arranged tertiary and quaternary rachises, uniovulate cupules and synangiate pollen organs terminating anisotomous fertile rachises. We tentatively reconstructed *Cosmosperma* as shown in Fig. 8, and it is thus one of the best morphologically understood Late Devonian seed plants in the world.

### Comparisons with other Devonian seed plants

The cupules and synangiate pollen organs of *Cosmosperma* have been compared with those of related seed plants [3], and this comparison section primarily focuses on frond morphology. Vegetative fronds have been reported in the Late Devonian seed plants, i.e., *Elkinsia* from USA [5], *Laceya* from Ireland [17, 18], *Kongshania* [8], *Yiduxylon* [13] and *Telangiopsis* [10] from China. Among them, *Elkinsia*, *Kongshania* and *Telangiopsis* are also known for fertile rachises with terminal pollen organs. Some selected morphological traits of these plants are listed and compared in Table 1. All of these taxa except *Kongshania* display bipartite fronds, while *Elkinsia* exhibits repeatedly bifurcated frond rachises exclusively. *Yiduxylon*, *Telangiopsis* and *Cosmosperma* have highly dissected, planated pinnules, differing from the laminate pinnules of *Elkinsia* and *Kongshania*. The fertile rachises bearing pollen organs are anisotomously divided in *Cosmosperma*, which enables them to be distinguished from the isotomously divided ones in *Elkinsia*, *Kongshania* and *Telangiopsis*. Prickles are extensively distributed on *Cosmosperma*, but are confined to the stems of *Telangiopsis* and absent from other coeval seed plants.

**Table 1:** Comparison of morphological traits among Late Devonian seed plants

Taxon	Frond rachis arrangement	Length of primary rachis prior to bifurcation (cm)	Location of ultimate pinnae	Pinnules	Fertile rachises bearing pollen organs	Prickles
*Elkinsia* [5]	Equally and repeatedly bifurcated	0-13^a^	on secondary and higher orders of rachises	Laminate	Isotomously and cruciately dichotomized	absent
*Laceya* [17, 18]	Pinnate with bifurcated primary rachis	up to 15.5	on both primary and secondary rachises	—	—	absent
*Kongshania* [8]	Pinnate	—	on tertiary rachises	Laminate	Isotomously dichotomized	absent
*Yiduxylon* [13]	Pinnate with bifurcated primary rachis	ca. 3	on tertiary rachises	Planate and highly dissected	—	absent
*Telangiopsis* [10]	Pinnate with bifurcated primary rachis	0^a^	on secondary rachises	Planate and highly dissected	Isotomously dichotomized	on stems
*Cosmosperma*	Pinnate with bifurcated/trifurcated primary rachis	10.3-21.2	on tertiary rachises	Planate and highly dissected	Anisotomously dichotomized	on stems, frond rachises and cupule surfaces

^a^: 0 stand for the basally bifurcated primary rachises

### Variations in fronds among early seed plants

Early seed plants are characterized by bipartite fronds with dichotomized primary rachises [19, 20], while diversified frond structures are evidenced in the Late Devonian taxa, such as variable dimensions of fronds, different branching manners and flexible locations of ultimate pinnae (Table 1). It has been shown that great morphological disparities have occurred among the Late Devonian spermatophytes. Lyginopterid seed plants in the Early/Late Carboniferous are thought to possess fronds with dichotomized/pinnate branching patterns, respectively [19]. Since *Elkinsia* is characterized by repeatedly dichotomized fronds [5], while *Laceya* [17], *Yiduxylon* [13], *Telangiopsis* [10] and *Cosmosperma* show pinnate fronds, it seems that both branching patterns have arisen in the Late Devonian spermatophytes.

The fertile fronds with terminal pollen organs often exhibit cruciate dividing patterns in the Late Devonian seed plants (e.g., *Telangium schweitzeri* [6] and *Elkinsia* [5]). Among the Early Carboniferous spermatophytes, the fertile fronds with terminal pollen organs containing trilete prepollen are divided into three types: *Rhacopteris*/*Triphyllopteris*-type, *Diplopteridium*-type and *Rhodea*-type [21]. The *Diplopteridium*-type illustrates a trifurcate frond rachis producing a median fertile rachis that is short and dichotomous [21–23]. The trifurcate fronds of *Cosmosperma* display a unique architecture among coeval seed plants. Such fronds, if connected to the fertile rachises bearing terminal pollen organs (Fig. 8), would greatly resemble the *Diplopteridium*-type fertile frond. In this case, *Cosmosperma* exemplifies the diversification of fertile fronds among Late Devonian seed plants, and suggests that some Carboniferous fertile frond types may be traced back to an earlier time.

Different dividing patterns of the fertile and vegetative fronds were present in Carboniferous spermatophytes [22, 23], which is also supported by the anatomical evidence [24, 25]. Both *Elkinsia* [5] and *Cosmosperma* indicate that the dimorphic fronds have occurred in the Late Devonian.

### Implications from the ovule of *Cosmosperma*

Nearly all Late Devonian seed plants have cupulate ovules (ovules enclosed in cupules) [2], and the cupules are uniovulate or multiovulate [4]. The uniovulate cupules were considered to be derived from the multiovulate ones [26, 27]. The uniovulate cupule has been proposed [3] and is now confirmed in *Cosmosperma*. Other Devonian seed plants with uniovulate cupules include *Dorinnotheca* [27], *Latisemenia* [4], *Condrusia* [28] and *Pseudosporognites* [2]. Their traits are listed in Table 2. The cupule or integument of the early ovules is considered archaic with numerous, terete and little fused segments or lobes [27, 29, 30]. In this case, *Cosmosperma* appears primitive among the ovules with uniovulate cupules.

**Table 2:** Comparison of Late Devonian seeds with uniovulate cupules

Taxon	Number of cupule segments	Structure of cupule segments	Number of cupule tips	Number of integumentary lobes	Shape and fusion of integumentary lobes
*Dorinnotheca* [27]	8	distally dissected	>40	4	Triangular, basally fused
*Condrusia* [28]	2	flattened and broad	2	—	—
*Pseudosporogonites* [2]	1	short, fused and collar/trumpet shaped	—	3-4	Flattened, 1/3 fused
*Latisemenia* [4]	5	broad and cuneate	5	4	Flattened, 1/2-2/3 fused
*Cosmosperma*	2?	Distal 1/2-2/3 dissected	up to 16	4	Linear, basal 1/3 fused

One of the most obvious functions of cupules and integuments is protection for the ovule [1], and a more entire (large and/or widely fused) integument may provide additional protection against water loss [4, 30]. The cupules of *Cosmosperma* enclose the ovule, while those of *Dorinnotheca*, *Pseudosporognites* and *Latisemenia* are recurved or short to extensively expose the ovule. On the other hand, the integrity of the integument is the lowest in *Cosmosperma*, moderate in *Dorinnotheca* and *Pseudosporognites*, and the greatest in *Latisemenia*. Therefore, the protection is largely provided by the cupule in *Cosmosperma*, and by the integument in the other three plants. The evolutionarily primitive status of *Cosmosperma* suggests that the protective function of uniovulate cupules may be replaced by the increasingly developed integument.

### Function of prickles and probable growth habit of *Cosmosperma*

The acute outgrowths of epidermis or both epidermis and cortex, without vascular tissues, are usually named prickles, while the sharp-pointed vascularized protuberances modified from axes and leaves are separately called thorns and spines [31, 32]. Commonly, the thorns and spines are only distributed along the axes and, owing to their internal vascular tissues, cannot be easily removed. However, in *Cosmosperma*, the tiny conical structures occur on stems, vegetative and fertile rachises and even cupules. They also present a highly variable density corresponding to loss in the transport and/or burial process. Therefore, we tentatively assign such structures to prickles.

The prickles are not common in the Late Devonian spermatophytes, but they have been reported in some later Paleozoic seed plants, including the Early Carboniferous *Medullosa steinii* and Late Permian gigantopterid *Aculeovinea yunguiensis* [33, 34]. It has been suggested that prickles on the cupule surface of *Cosmosperma* may serve as protection [3]. On the other hand, arthropod herbivory was recorded in some Late Devonian myriapods and apterygote hexapods [35], while the major plant defensive adaptations to such herbivory are considered chemical [36]. However, the terrestrial vertebrate herbivory did not occur until the Permian [34]. Since prickles are considered to provide mechanical attachments in other younger Paleozoic seed plants [33, 34], it is plausible that the prickles on the axes and leaves of the Late Devonian seed plants may largely function as supporting structures rather than defense structures against the herbivores.

Previous studies have suggested that the seed plants assigned to the Lyginopteridales are vines/lianas possessing stems generally less than 20 mm wide, and those to the Calamopityales are upright with stems usually over 20 mm wide [13, 25]. Other evidence that supports lyginopterids as vines/lianas includes stems bearing long internodes, the presence of adventitious roots, large fronds with swollen frond bases, wide angle of frond attachment and *Dictyoxylon*-type outer cortex [13, 37, 38]. *Cosmosperma* possesses relatively large fronds with pulvinus-shaped bases, which resemble those of lyginopterids. The extensively born prickles of *Cosmosperma* also remind us of the glands on *Lagenostoma* and *Lyginodendron* [39]. However, in *Cosmosperma*, the width of the stems reaches 22 mm, the internodes are relatively short, the adventitious root is absent, the fronds depart at 40–70° and the cortex is most likely *Sparganum*-type. These traits enable *Cosmosperma* to be tentatively reconstructed as an upright, probably semi-self-supporting plant (Fig. 8), which may support each other by entangled bushy fronds rather than scrambling or climbing. The hypothesis is supported by the preservation that many slabs exhibit pure and dense communities of *Cosmosperma*, without any other arborescent plants. The prickles may help anchor fronds of adjacent individuals. However, the anatomical information is needed to test the suggested growth habits of this plant.

## Conclusions

We further studied the seed plant *Cosmosperma polyloba* from the Upper Devonian of South China, and its stems, fronds, cupulate ovules and fertile rachises bearing pollen organs are now known in detail. Based on the morphological evidence mentioned above, we tentatively reconstructed the whole plant with an upright, semi-self-supporting habit. The prickles on stems and rachises may facilitate supporting. The fronds of *Cosmosperma* show bifurcated or trifurcated primary rachises, which further add to the diversity and demonstrate dimorphism of the early spermatophyte fronds. The less-fused cupules and integuments suggest that *Cosmosperma* is primitive among Late Devonian seed plants with uniovulate cupules.

## Additional files

Additional file 1: Figure S1. Interpretative line drawings showing branching pattern of *Cosmosperma polyloba*. Abbreviations: st, stem; pr, primary rachis; sr, secondary rachis; tr, tertiary rachis. (a) Stem, primary and secondary rachises and basal part of a tertiary rachis in Fig. 1h. (b) Bifurcate primary rachis, two secondary rachises, and a tertiary rachis bearing ultimate pinnae and conical prickles in Fig. 6c. (c) Secondary rachis with alternate tertiary rachises, ultimate pinnae and conical prickles in Fig. 6b. (d) Bifurcated primary rachis, two secondary rachises and alternate tertiary rachises with ultimate pinnae and conical prickles in Fig. 4a. (TIFF 2282 kb)

Additional file 2: Figure S2. Interpretative line drawings showing frond and ultimate pinnae of *Cosmosperma polyloba*. Abbreviations same as in Figure S1. (a) Bifurcate primary rachis, two secondary rachises, and one tertiary rachis with 11 ultimate pinnae in Fig. 6a. (b-e) Ultimate pinnae in Fig. 6(c, left arrow), Fig. 6(c, right arrow), Fig. 6(b, arrow) and Fig. 4(c), respectively. Highly dissected and planate pinnules alternately arranged along the quaternary rachis. (TIFF 2025 kb)

Additional file 3: Figure S3. Interpretative line drawing showing synangiate pollen organs on fertile axes of *Cosmosperma polyloba*. (a) Anisotomous fertile rachises with terminal pollen organs in Fig. 7a. Conical prickles sparsely located along the fertile rachises sparsely. (b, c) Two stages of dégagement on pollen organs in Fig. 7d, e, respectively. (TIFF 1395 kb)
