# Supplementary figures and images for: Further study of Late Devonian seed plant Cosmosperma polyloba: its reconstruction and evolutionary significance
Source: BMC Evol Biol. 2017 Jun 26;17:149. doi: 10.1186/s12862-017-0992-1 (PMC5485708; doi:10.1186/s12862-017-0992-1)

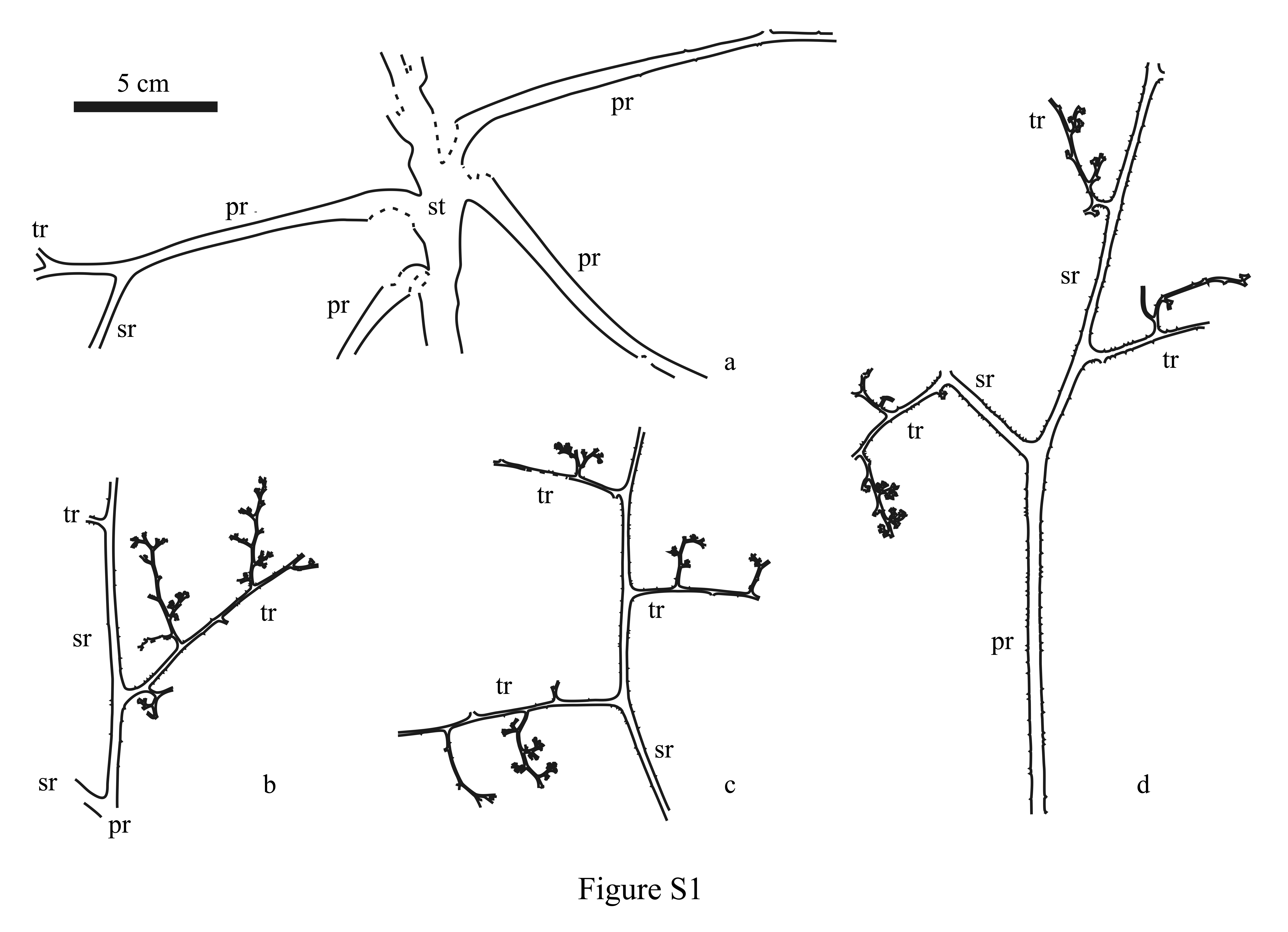

Supplement: Supplementary file 1 — Interpretative line drawings showing branching pattern of Cosmosperma polyloba. Abbreviations: st, stem; pr, primary rachis; sr, secondary rachis; tr, tertiary rachis. (a) Stem, primary and secondary rachises and basal part of a tertiary rachis in Fig. 1h. (b) Bifurcate primary rachis, two secondary rachises, and a tertiary rachis bearing ultimate pinnae and conical prickles in Fig. 6c. (c) Secondary rachis with alternate tertiary rachises, ultimate pinnae and conical prickles in Fig. 6b. (d) Bifurcated primary rachis, two secondary rachises and alternate tertiary rachises with ultimate pinnae and conical prickles in Fig. 4a. (TIFF 2282 kb) [file 12862_2017_992_MOESM1_ESM.tif]

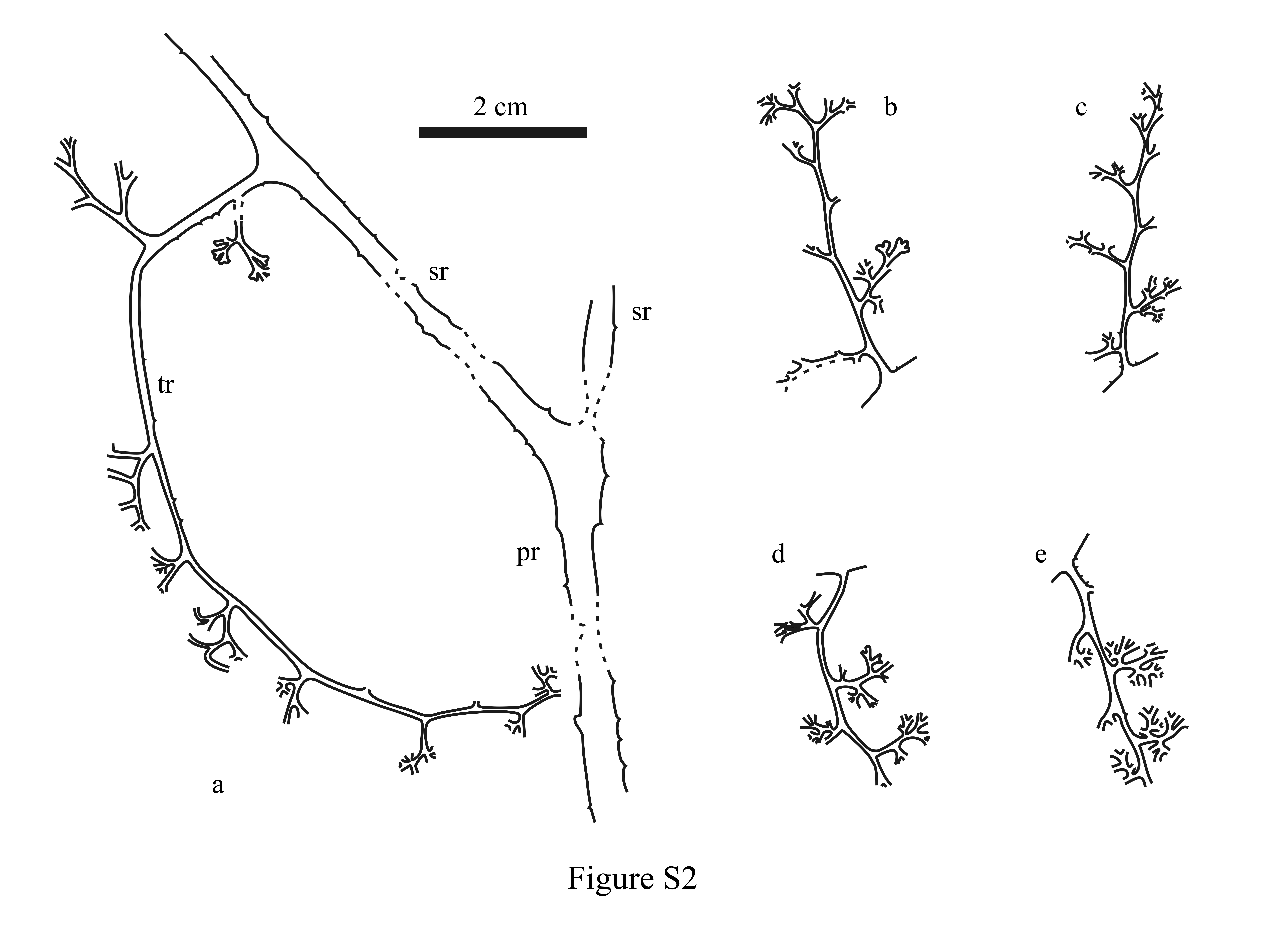

Supplement: Supplementary file 2 — Interpretative line drawings showing frond and ultimate pinnae of Cosmosperma polyloba. Abbreviations same as in Figure S1. (a) Bifurcate primary rachis, two secondary rachises, and one tertiary rachis with 11 ultimate pinnae in Fig. 6a. (b-e) Ultimate pinnae in Fig. 6(c, left arrow), Fig. 6(c, right arrow), Fig. 6(b, arrow) and Fig. 4(c), respectively. Highly dissected and planate pinnules alternately arranged along the quaternary rachis. (TIFF 2025 kb) [file 12862_2017_992_MOESM2_ESM.tif]

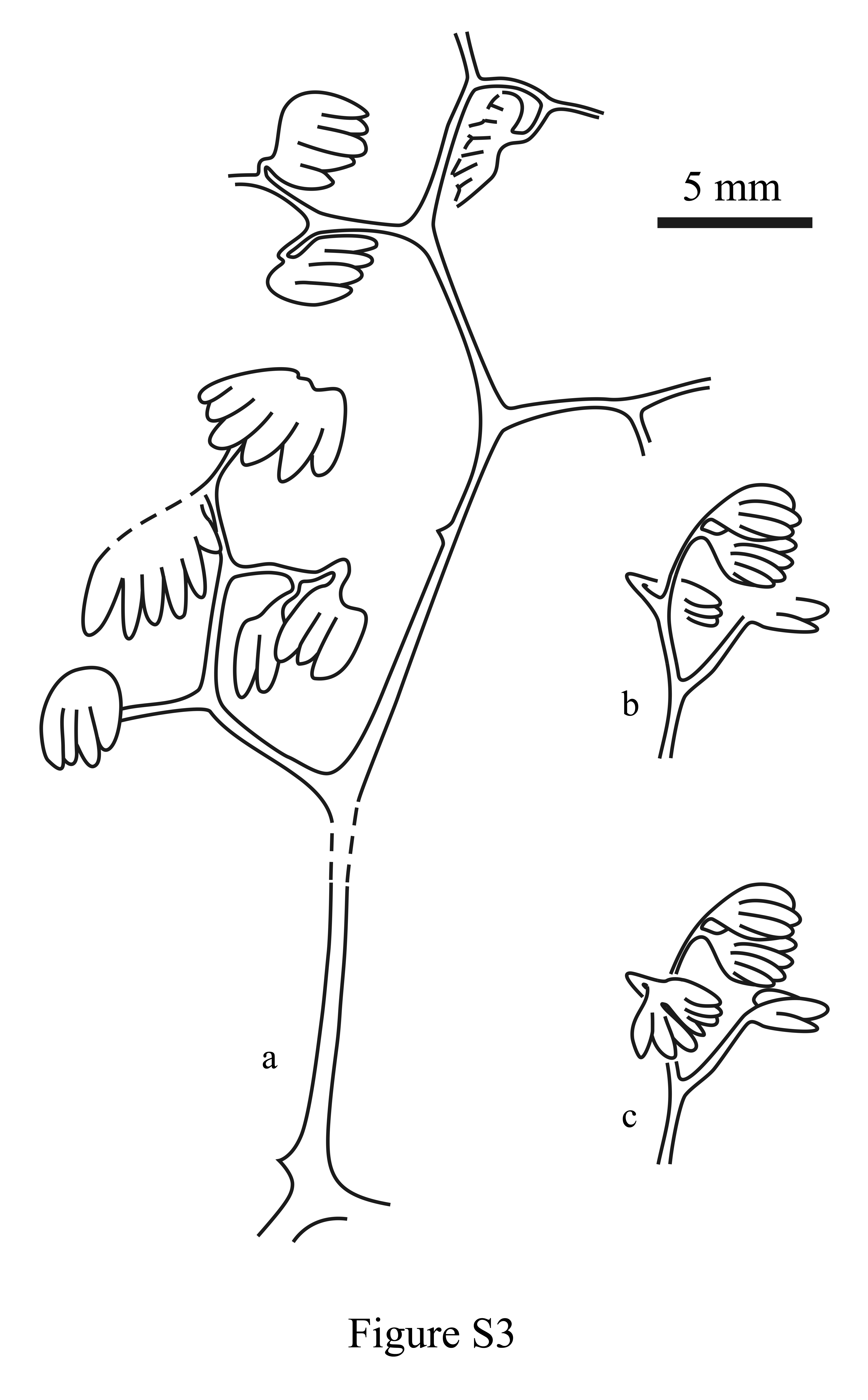

Supplement: Supplementary file 3 — Interpretative line drawing showing synangiate pollen organs on fertile axes of Cosmosperma polyloba. (a) Anisotomous fertile rachises with terminal pollen organs in Fig. 7a. Conical prickles sparsely located along the fertile rachises sparsely. (b, c) Two stages of dégagement on pollen organs in Fig. 7d, e, respectively. (TIFF 1395 kb) [file 12862_2017_992_MOESM3_ESM.tif]
